# U.S. state policy contexts and mortality of working-age adults

**DOI:** 10.1371/journal.pone.0275466

**Published:** 2022-10-26

**Authors:** Jennifer Karas Montez, Nader Mehri, Shannon M. Monnat, Jason Beckfield, Derek Chapman, Jacob M. Grumbach, Mark D. Hayward, Steven H. Woolf, Anna Zajacova

**Affiliations:** 1 Department of Sociology, Syracuse University, Syracuse, NY, United States of America; 2 Aging Studies Institute, Syracuse University, Syracuse, NY, United States of America; 3 Department of Sociology, Harvard University, Cambridge, MA, United States of America; 4 Division of Epidemiology, Virginia Commonwealth University, Richmond, VA, United States of America; 5 Department of Political Science, University of Washington, Seattle, WA, United States of America; 6 Department of Sociology, University of Texas at Austin, Austin, TX, United States of America; 7 Department of Family Medicine and Population Health, Virginia Commonwealth University, Richmond, VA, United States of America; 8 Department of Sociology, University of Western Ontario, Ontario, CA, United States of America; Johannes Gutenberg Universitat Mainz, GERMANY

## Abstract

The rise in working-age mortality rates in the United States in recent decades largely reflects stalled declines in cardiovascular disease (CVD) mortality alongside rising mortality from alcohol-induced causes, suicide, and drug poisoning; and it has been especially severe in some U.S. states. Building on recent work, this study examined whether U.S. state policy contexts may be a central explanation. We modeled the associations between working-age mortality rates and state policies during 1999 to 2019. We used annual data from the 1999–2019 National Vital Statistics System to calculate state-level age-adjusted mortality rates for deaths from all causes and from CVD, alcohol-induced causes, suicide, and drug poisoning among adults ages 25–64 years. We merged that data with annual state-level data on eight policy domains, such as labor and taxes, where each domain was scored on a 0–1 conservative-to-liberal continuum. Results show that the policy domains were associated with working-age mortality. More conservative marijuana policies and more liberal policies on the environment, gun safety, labor, economic taxes, and tobacco taxes in a state were associated with lower mortality in that state. Especially strong associations were observed between certain domains and specific causes of death: between the gun safety domain and suicide mortality among men, between the labor domain and alcohol-induced mortality, and between both the economic tax and tobacco tax domains and CVD mortality. Simulations indicate that changing all policy domains in all states to a fully liberal orientation might have saved 171,030 lives in 2019, while changing them to a fully conservative orientation might have cost 217,635 lives.

## Introduction

Americans die younger than people in most other high-income countries. With a life expectancy of 78.8 years in 2019, Americans died 5.7 years earlier than people in Japan, the global leader; 3.3 years earlier than their northern neighbors in Canada; and 2.5 years before their closest geopolitical allies in the United Kingdom [1]. Shockingly, U.S. life expectancy falls between two middle-income countries—Cuba and Albania [1]. Within the United States, life expectancy differs markedly across geographic areas such as states and counties. In 2019, it ranged from 74.4 years in Mississippi to 80.9 years in Hawaii [2].

U.S. life expectancy has stagnated, largely because of higher mortality among adults 25–64 years of age [3–6]. According to a comparison of U.S. life expectancy to the average of 16 other high-income countries in 2006–2008, deaths before age 50 accounted for 67% of the shortfall among U.S. men and 41% among women [3]. Mortality rates provide another sobering picture of the early deaths among so many individuals in the United States. Based on rates from 2019, for every 100 babies born in the United States, two will not survive to their 30^th^ birthday, six will not reach age 50, and 16 will die before they can enjoy retirement at age 65 [7]. Like life expectancy at birth, differences across states in mortality rates among adults ages 25–64 are striking [4, 6].

Several studies have examined the causes of death contributing to the high and rising mortality rates of U.S. adults. An analysis of the plateau in U.S. life expectancy at age 25 that occurred between 2010 and 2017 identified the slowing decline in cardiovascular disease (CVD) mortality as the dominant cause [8, 9]. A 2021 National Academies of Sciences, Engineering, and Medicine (NASEM) report implicated cardiometabolic causes of death, especially CVD, along with deaths from alcohol-induced causes, suicide, and drug poisonings as major causes of rising working-age (25–64 years) mortality during 1990–2017 [4]. Several of these causes are also implicated in the U.S. mortality disadvantage relative to other countries. One comparison of U.S. life expectancy in 2006–2008 with that of other high-income countries attributed the higher U.S. death rate to noncommunicable diseases (especially CVD), unintentional injuries, and homicide (for men); the higher death rate before age 50 was driven by noncommunicable diseases, unintentional injuries (mostly drug poisonings), intentional injuries (e.g., suicide), and perinatal conditions [3]. A more recent international analysis of 2014–2015 found that higher U.S. death rates before age 65 were due to drugs, suicide, and homicide, plus higher CVD and respiratory mortality among women [10]. In sum, evidence consistently points to CVD, drug poisoning, suicide, and alcohol-induced deaths as key drivers of high mortality rates among working-age Americans.

No single factor has been identified to explain the increase in U.S. mortality or the U.S. disadvantage compared with peer countries; the root causes are complex and multifaceted. The 2021 NASEM report advanced a helpful, multilayered conceptual framwork that recognizes influences on mortality at three levels: *macro*-level (e.g., social, political, and cultural structures, among them state and federal policies), *meso*-level (e.g., community, family, and work structures), and *individual*-level (e.g., proximate factors like behaviors, biology, and socioeconomic status) [4]. The report underscored the importance of the macro layer, as it influences the other two layers. For instance, U.S. state policies can shape individuals’ economic circumstances (e.g., via minimum wage levels and earned income tax credits), family conditions (e.g., via paid leave and reproductive health care), environmental conditions (e.g., via policies on housing, food deserts, green space, pollution, and climate change), and behaviors (e.g., via tobacco taxes and marijuana legalization).

A growing number of scientists have pushed for greater attention to macro explanations to better understand the factors driving the high, rising, and unequal mortality rates among U.S. adults [11–19]. Bambra and colleagues asserted that, “to properly understand the U.S. mortality disadvantage, research needs to ‘scale up’ and refocus on upstream political, economic, and policy drivers” [12(p. 39)]. Montez urged researchers to ‘hypothesize upward’ and shine a light on the growing importance of U.S. state policy contexts over the last 40 years [14]. In fact, emerging evidence suggests that U.S. policy and political contexts have had detrimental consequences [13, 19, 20]. One study found that U.S. life expectancy could increase by nearly four years if the country matched the average level of social policy generosity offered in 17 other high-income countries [13]. More recent research has turned attention to policies and politics at the U.S. state level, given the federalist structure of the U.S. political system and the large size and geographical spread of the population [14–16]. This new work suggests that changes in state policies and politics may have played a contributory role in producing the troubling U.S. mortality trends, as highlighted in the next section.

### U.S. state policy contexts

**Fig 1:**
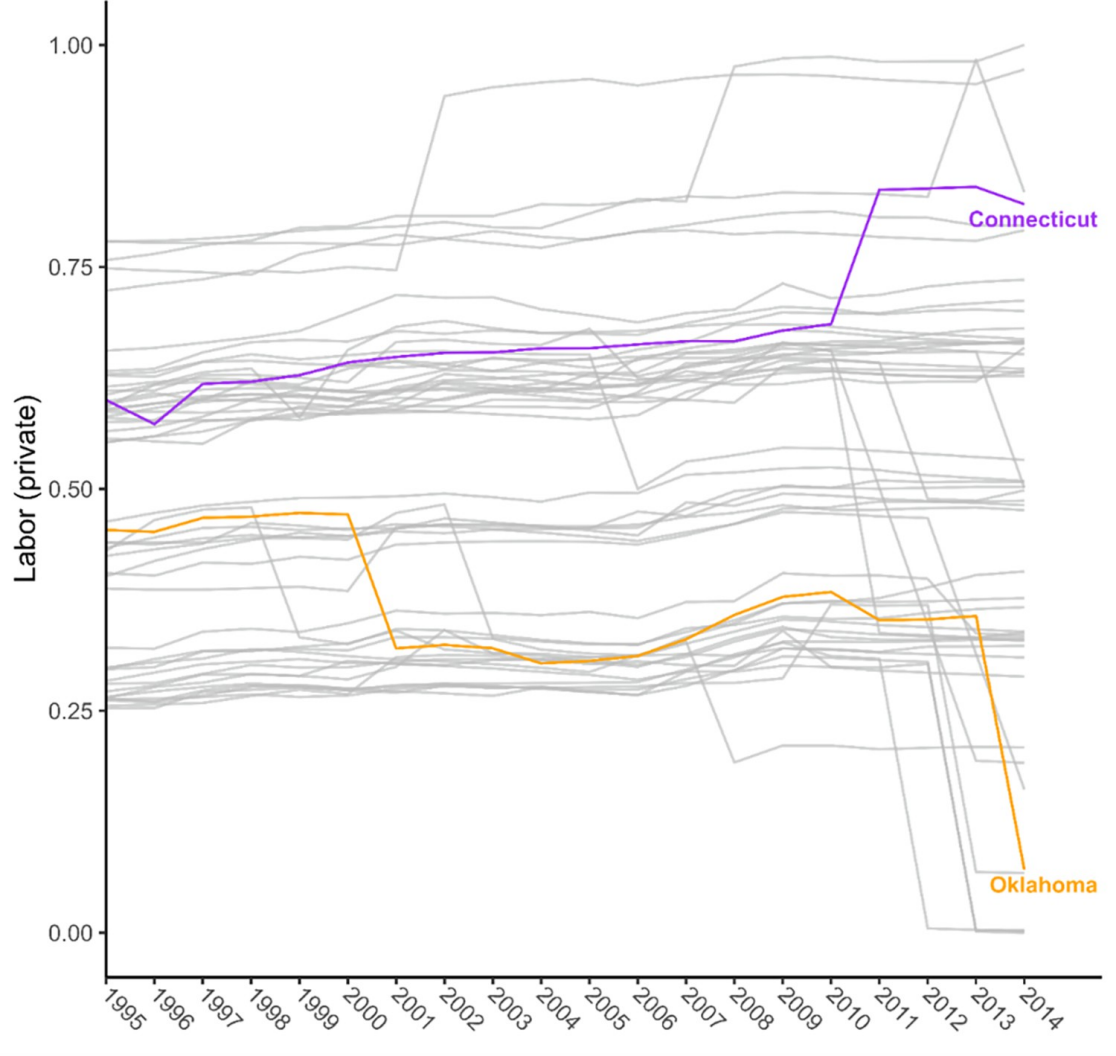
Trends in liberalism scores for private labor policies by U.S. State, 1995–2014. The figure contains a line for every state. Scores range from 0 to 1 on a conservative to liberal continuum.

For approximately 40 years, the policy contexts in which Americans live have been increasingly defined by their state of residence [21]. State policies have hyperpolarized during this period, with many states’ policy contexts moving toward more extreme “left” or “right” positions on the political spectrum [15, 21, 22]. One consequence has been growing policy divergence across states. Today, some states’ policy contexts emphasize investments in education, economic stability for working adults provided through higher minimum wages and earned income tax credits, the discouragement of risky health behaviors through excise taxes (e.g., on tobacco products), expanded access to affordable health care, and more. In contrast, many other states’ policy contexts have moved in the opposite direction, particularly on policies concerning labor, guns, abortion, and the environment [21]. As an example, Fig 1 shows the diverging trends in U.S. states’ labor policies in recent decades, contrasting Connecticut and Oklahoma. Created by Grumbach [21], the annual labor policy score summarizes multiple labor policies (e.g., minimum wage, right-to-work laws, unemployment insurance) in each state in each year on a 0–1 conservative-to-liberal continuum.

These tectonic political and policy shifts may have had profound impacts on health and wellbeing. In fact, a recent study documented an association between state policy contexts and life expectancy at birth; overall changes in those contexts since the 1980s appear to have suppressed gains in life expectancy, particularly after 2010 [20]. The study combined annual data on states’ policy domains, such as labor and environment, with annual data on states’ life expectancies from 1970 through 2014. Each domain captured multiple specific polices. For example, the labor domain included nine policies, such as minimum wage levels, disability insurance, and right-to-work laws. Each domain was scored to reflect how liberal or conservative a state’s policies were on that domain in each year [21]. The study found that several policy domains—notably those on the environment, civil rights, tobacco, labor, and immigration—were key predictors of life expectancy [20]. Except for marijuana policy, more liberal versions of the policy domains predicted higher life expectancy. The study also estimated that the rate of growth in U.S. life expectancy during the last five years of the study (2010–2014) would have been 13% steeper for men and 25% steeper for women if state polices had not changed in the way they did.

The above study raised several important and underexplored questions about the potential role of U.S. state policy contexts on the high and rising rates of working-age mortality. For example, does the association between state policies and life expectancy extend to working-age mortality? If so, how much higher or lower might working-age mortality rates have been if policy contexts had been different, all else being equal? Also unclear is how state policy contexts shape the risks of dying from specific causes such as CVD. Are some policies associated with multiple causes of death, indicating a broad influence on multiple disease and injury domains, and are other policies associated with only a few causes of death? To what extent do the observed associations suggest potential causal mechanisms or confounding variables? Are some policies associated with lower mortality from some causes of death but higher mortality from other causes, suggesting countervailing influences on working-age mortality? Answers to these questions may help explain the high mortality rates among working-age adults, identify policy domains that could potentially reduce those rates, and, by examining cause-specific mortality, provide insights on possible mechanisms linking state policies to working-age mortality.

### Aims

This study’s main aim is to understand how state policy contexts in recent decades are associated with working-age mortality overall and from CVD, alcohol-induced causes, suicide, and drug poisoning. The study centers on two main questions. First, how were state policy contexts associated with working-age mortality in recent decades? We analyze a subset of the policy domains used in the recent study of state policies and life expectancy [20, 21]. In addition, we assess how robust the findings are to various lag times between changes in the policy domains within a state and changes in mortality within a state. Second, how might hypothetical changes in state policy contexts affect working-age mortality? We simulate the mortality consequences of four hypothetical policy contexts. For both questions, we focus on adults ages 25–64 years and conduct the analyses separately by sex. The findings provide new insights into how state policy contexts are associated with working-age mortality and how changing those contexts might save lives.

## Materials and methods

### Mortality data

Mortality data come from the restricted Multiple Cause-of-Death files, accessed through an agreement with the National Center for Health Statistics. The data identify the underlying cause of death and state of residence from all death certificates filed from 1999 through 2019. We calculated annual, age-adjusted (to the 2000 U.S. population) mortality rates for adults ages 25–64 years by state, separately for males and females. We calculated rates for all-cause mortality and mortality from CVD (ICD-10 codes I00-I99), alcohol-induced causes (E24.4, F10, G31.2, G62.1, G72.1, I42.6, K29.2, K70, K85.2, K86.0, R78.0, X45, X65, Y15), suicide (X66-X84, Y87.0), and drug poisonings (X40-X44, X60-X64, X85, Y10-Y14). The external cause categories (alcohol-induced causes, suicide, and drug poisonings) are mutually exclusive: for example, suicides by drugs are included in drug poisoning deaths, and suicides by alcohol are included in alcohol-induced deaths. We focused on these four causes of death because, as we described above, stagnating declines in CVD mortality, alongside increases in deaths from alcohol-induced causes, suicide, and drug poisonings, are among the most important contributors to increasing working-age mortality in recent decades.

### State policy data

Data on state policies come from Grumbach [21], who collected annual information (from 1970 through 2014) on 135 policies from a variety of public sources [23] and grouped them into 16 domains: abortion, campaign finance, civil rights and liberties, criminal justice, marijuana, education, environment, gun safety, health and welfare, housing and transportation, immigration, private sector labor, public sector labor, LGBT, economic taxes, and voting. A few of the 135 policies were placed in an “other” domain. From this domain, we included tobacco excise taxes (in 2015 dollars), given their role in deterring smoking, the leading preventable cause of death [24, 25].

Using an established methodology [26], Grumbach assigned a policy liberalism score from 0 to 1 to each of the 16 policy domains, for every state-year observation, where the 0–1 range represents a conservative-to-liberal continuum [21]. Policies were considered liberal if they expand state power for economic regulation and redistribution, protect the rights of marginalized groups, or restrict state power to punish deviant behavior. Policies with opposite aims were considered conservative. The 0–1 scores represent the actual range during the 1970–2014 period. As an example, a score of 0 on the environmental domain for a particular state in a particular year means that state in that year had the most conservative configuration of environmental policies across all states and years. We normalized tobacco excise taxes on a 0–1 scale across state-year observations.

This study examined eight of the 16 policy domains that met two inclusion criteria. These eight domains (1) have been shown in, or strongly suggested by, existing studies to influence working-age mortality, especially from the causes of death that we examine, and (2) exhibited adequate within-state variation over time during our study period (trends in each domain are shown in S1 Fig in S1 File and in Grumbach [21]). Temporal variation within states is needed for the regression models, as described below. Some domains exhibited temporal variation but lacked existing evidence for a mortality effect (e.g., campaign finance), while others were reasonably justified in the literature but varied little during the period (e.g., public labor). Eight domains met both criteria: criminal justice, marijuana, environment, gun safety, health and welfare, private labor, economic taxes, and tobacco taxes. The policies included in these domains are in Table 1.

**Table 1:** Policies included in the eight domains (adapted from Grumbach 2018).

**Criminal Justice**	**Health and Welfare**
Death penalty repeal	Affordable Care Act (ACA) exchange
Determinate sentencing	AFDC payment level
DNA motions	AFDC Up
Three strikes	CHIP eligibility (children)
Truth-in-sentencing	CHIP eligibility (infants)
	CHIP eligibility (pregnant women)
**Economic Taxes**	Expanded dependent coverage
Corporate tax rate	Medicaid adoption
Earned income tax credit	Medicaid expansion
Estate tax	Pre- Balanced Budget Act CHIP eligibility
Income tax	Senior prescription drugs
Sales tax	TANF eligibility
Tax burden	TANF payment level
Top capital gains rate	Welfare drug test
Top income rate	Welfare time limit
**Environment**	**Marijuana**
Bottle bill	Marijuana decriminalization
California car emissions standards	Medical marijuana
Endangered species	
E-waste	**Private Labor**
Greenhouse Gas (GHG) cap	Disability insurance
Renewables fund	Local minimum wage ban
Solar tax credit	Local sick leave law ban
State NEPA	Minimum wage
	Paid family leave
**Firearm Safety**	Paid sick leave
Assault weapon ban	Prevailing wage
Background checks (dealers)	Right to work
Background checks (private)	Unemployment compensation
Brady law	
Dealer licenses required	**Tobacco Tax**
Gun registration	State tax on a pack of cigarettes
Open carry	
Saturday night special ban	
Stand your ground	

We combined the policy and mortality datasets in several ways to evaluate lag times of one to five years, as it may take time to see an association between changes in a some policy domains and mortality rates (we did not lag tobacco tax). Recall that the policy data span 1970–2014, and the mortality data span 1999–2019. We first combined the policy and mortality datasets using a 0-year lag, such that each state’s policy data in year *Y* was merged with its mortality data in year *Y*. This dataset spans 1999 to 2014. We then combined the policy and mortality datasets using a 1-year lag such that each state’s policy data in year *Y* was merged with its mortality data in *Y+1*. This dataset spans 1999 to 2015 (policy data from 1998–2014 were merged into mortality data from 1999–2015). We repeated this process, creating additional datasets to evaluate 2, 3, 4, and 5-year lags. For example, the dataset for estimating models with a 5-year lag spans 1999 to 2019 (policy data from 1994–2014 were merged with mortality data spanning 1999–2019).

### Approach

We estimated sex-specific regression models of the form shown in Eq 1. The models regressed the log(age-adjusted mortality rate) in state *i* in year *j* on the policy domains *P*, time-varying state-level covariates *Z*, a linear measure of time *T* (alternative specifications of time did not improve the model or alter the findings), fixed effects for each state *α*_*i*_, and an error term *μ*_*ij*_.


$${log\;(age‐adjusted\;mortality\;rate)}_{ij}={\beta_{p}P_{ij}+\beta }_{z}Z_{ij}+\beta_{t}T_{j}+\alpha_{i}+\mu_{ij}$$ (1)

Several of the time-varying covariates came from work relating the domains to life expectancy [20]. One covariate was the annual proportion of each state’s population who were immigrants, given changes in the size of the immigrant population in some states during the study period and the known mortality advantage of first-generation immigrants [27]. As a proxy for the macroeconomic environment, we included each state’s annual unemployment rates, which can influence adult mortality [28, 29]. Lastly, following the approach of others [30], we included the annual number of each state’s laws on opioid regulation and harm mitigation, which were increasingly enacted during the latter years of our study period. The six areas of legislation in this measure include access to prescription drug monitoring programs (PDMP), mandatory PDMP, prescription limits, pain clinics, Good Samaritan laws, and naloxone access, with data taken from Lee et al. [31]. We did not include covariates that are pathways, or downstream determinants, through which state policies might affect mortality. For example, we do not adjust for education levels or income of states’ residents. Importantly, recent findings show that growing disparities in working-age mortality across states are not due to diverging education levels or income [32].

We included fixed effects for states to control for time-invariant observed or unobserved state-level characteristics. For instance, during the study period, Mississippi had a hotter climate and a greater percentage of its population was Black, rural, poor, and religious than Minnesota. Including fixed effects allows the models to estimate how within-state variation in mortality is associated with within-state variation in the policy domains. The models corrected for spatial and temporal correlations among the state-year observations using Driscoll and Kraay [33] standard errors with the xtscc command in Stata [34]. Lastly, like other studies, we weight observations by state population to provide nationally representative estimates and avoid inflating the influence of small states [20, 29, 30, 35, 36].

Using two decades of data, multiple policy domains, fixed effects for states, lagged analyses, and addressing several sources of confounding, this study is nevertheless not designed to assess whether the domains have a causal effect on mortality. Studies that identify causal effects often attempt to isolate the effects of *specific* policies, rather than the general liberal or conservative character of policy *domains*, holding other conditions constant. The policy domains used in this study do not easily lend themselves to causal interpretation, as they reflect *combinations* of policies. Nevertheless, the combinations are a strength for our objective to understand how multiple dimensions of states’ policies are associated with working-age mortality. They reflect “the reality that people live more than one policy at a time” [37(p 234)] and that policies are often implemented in bundles [38].

Our main and supplementary analyses contain many models, each of which assesses multiple policy domains. Given this complexity, we present most results graphically and focus on patterns, similarities, and contrasts across the models, rather than a particular coefficient, policy domain, or significance test. This strategy also helps avoid over-interpretation of specific results and reliance on null hypothesis testing. S1 File provides the detailed models results.

## Results

### All-cause mortality

**Fig 2:**
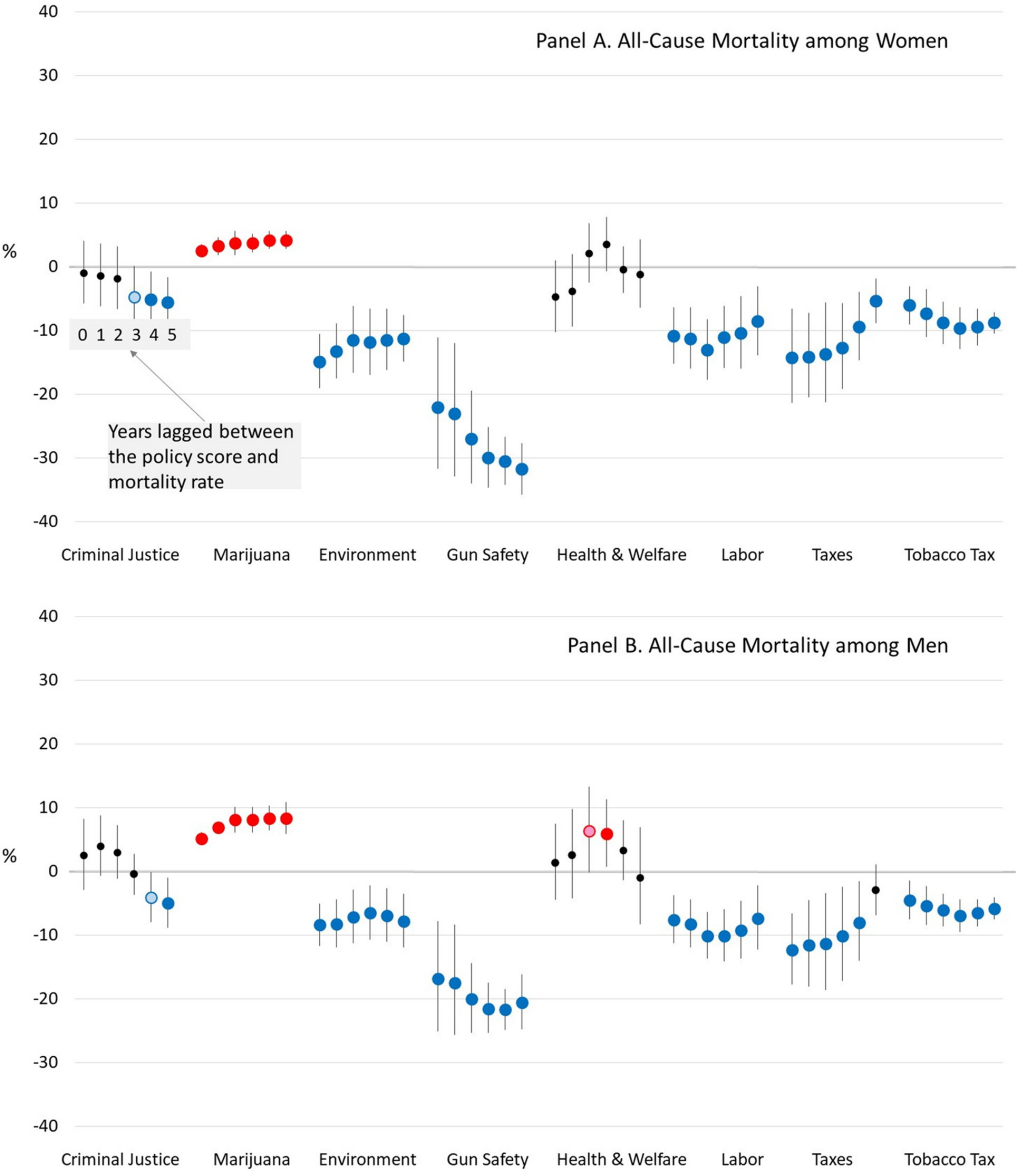
Estimated percent difference in all-cause mortality rates when a U.S. State’s policy liberalism score is 1 versus 0, for various lag times between the policies and mortality. *Notes*: Blue dots mean a more liberal version of the policy is associated with lower mortality and red dots mean a more conservative version is associated with lower mortality. Dark blue and red dots indicate that the association is significant at *α*<0.05, while light blue and red dots indicate that it is significant at *α*<0.10.

The results for women are displayed in the top panel of Fig 2. It summarizes the results of six models predicting working-age mortality, where each model uses a different lag time (from 0 to 5 years) and includes all eight policy domains, the covariates, time, and state fixed effects. For each policy domain, the figure presents the estimated difference in all-cause mortality rates among women ages 25–64 residing in a state with the most liberal policies in that domain (liberalism score = 1) versus the same state but with the most conservative policies (liberalism score = 0).

We illustrate the interpretation of the results using the criminal justice policy domain. Assuming no lag time, the all-cause mortality rate among women ages 25–64 years in a state with the most liberal criminal justice policies is estimated to be 0.9% lower (95% confidence interval [CI]: -5.7%, 4.1%) than if that same state had the most conservative policies, net of the other seven policy domains, the covariates, time, and state fixed effects. The association between criminal justice policies and women’s mortality increased with longer lag times and reached statistical significance with lag times of 3 years (*α*<0.10) and 4 and 5 years (*α*<0.05). Using a 5-year lag, all-cause mortality rates among working-age women were estimated to be 5.6% lower (95% CI: -9.4%, -1.7%) if a state had the most liberal criminal justice score than if that same state had the most conservative score, net of the other policies, covariates, time, and fixed effects.

Lower all-cause mortality rates among working-age women are also associated with higher liberalism scores for the environment, gun safety, labor, economic taxes, and tobacco tax domains, regardless of lag time. More liberal scores on the marijuana domain are associated with higher mortality rates, regardless of lag time. The magnitude of the associations is especially large for gun safety. Using a 5-year lag, women’s all-cause mortality was estimated to be 31.8% lower (95% CI: -35.7%, -27.6%) if a state had the most liberal gun safety policies than if it had the most conservative, net of other policies, covariates, time, and fixed effects. The results for men are shown in the bottom panel of Fig 2 and are generally similar.

### Cause-specific mortality

**Fig 3:**
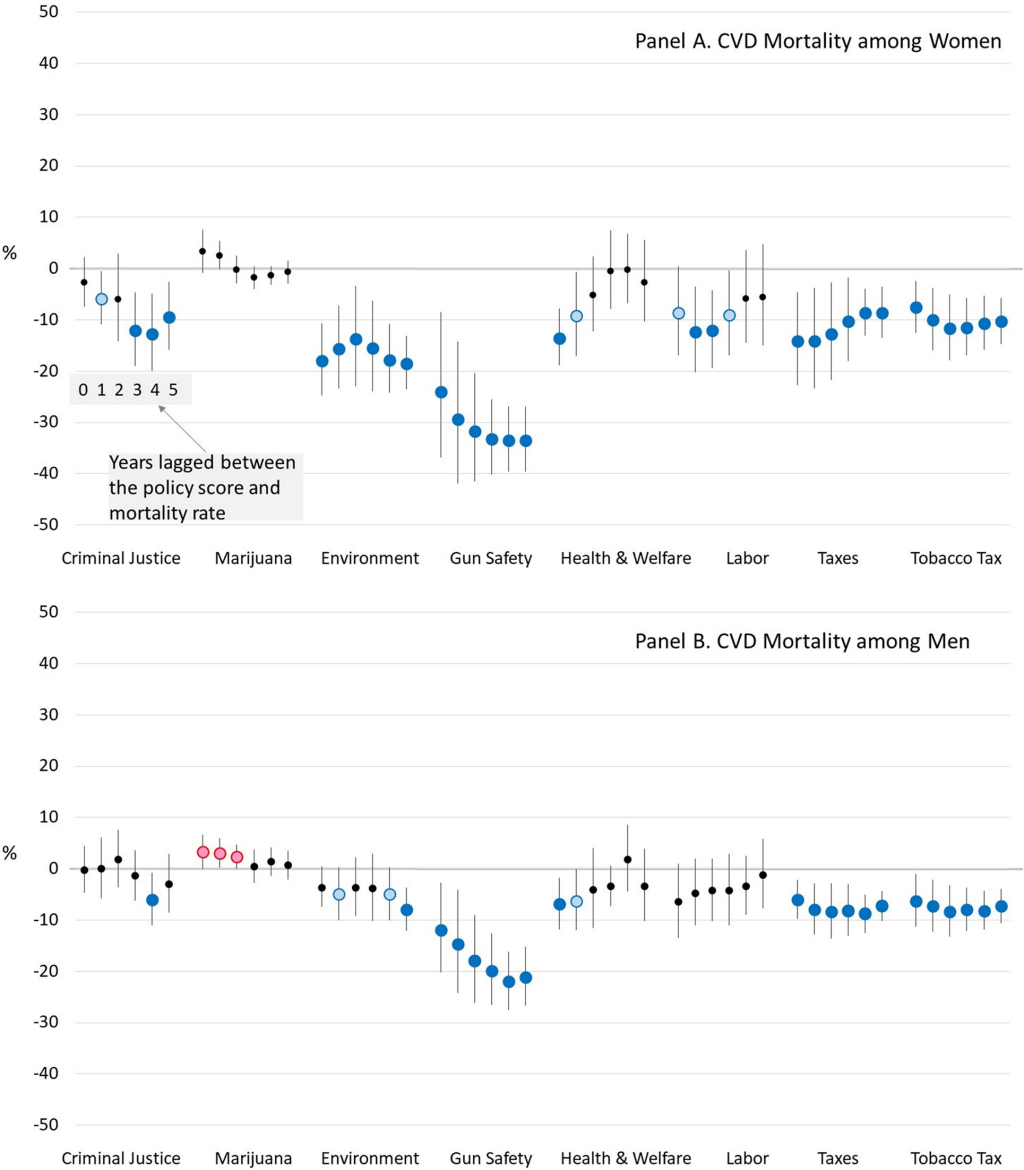
Estimated percent difference in cardiovascular mortality rates when a U.S. State’s policy liberalism score is 1 versus 0, for various lag times between the policies and mortality. *Notes*: Blue dots mean a more liberal version of the policy is associated with lower mortality and red dots mean a more conservative version is associated with lower mortality. Dark blue and red dots indicate that the association is significant at *α*<0.05, while light blue and red dots indicate that it is significant at *α*<0.10.

Fig 3 shows that several policy domains have significant associations with CVD mortality among adults ages 25–64 years. More liberal gun safety, economic tax, and tobacco tax policy domains were associated with lower CVD mortality in both women and men, regardless of lag time. More liberal environment policies predicted lower CVD mortality among women (and was suggested among men). More liberal criminal justice policies were associated with reduced CVD mortality among women for most lag times. In contrast to all-cause mortality, CVD mortality exhibited little association with the marijuana policy domain.

**Fig 4:**
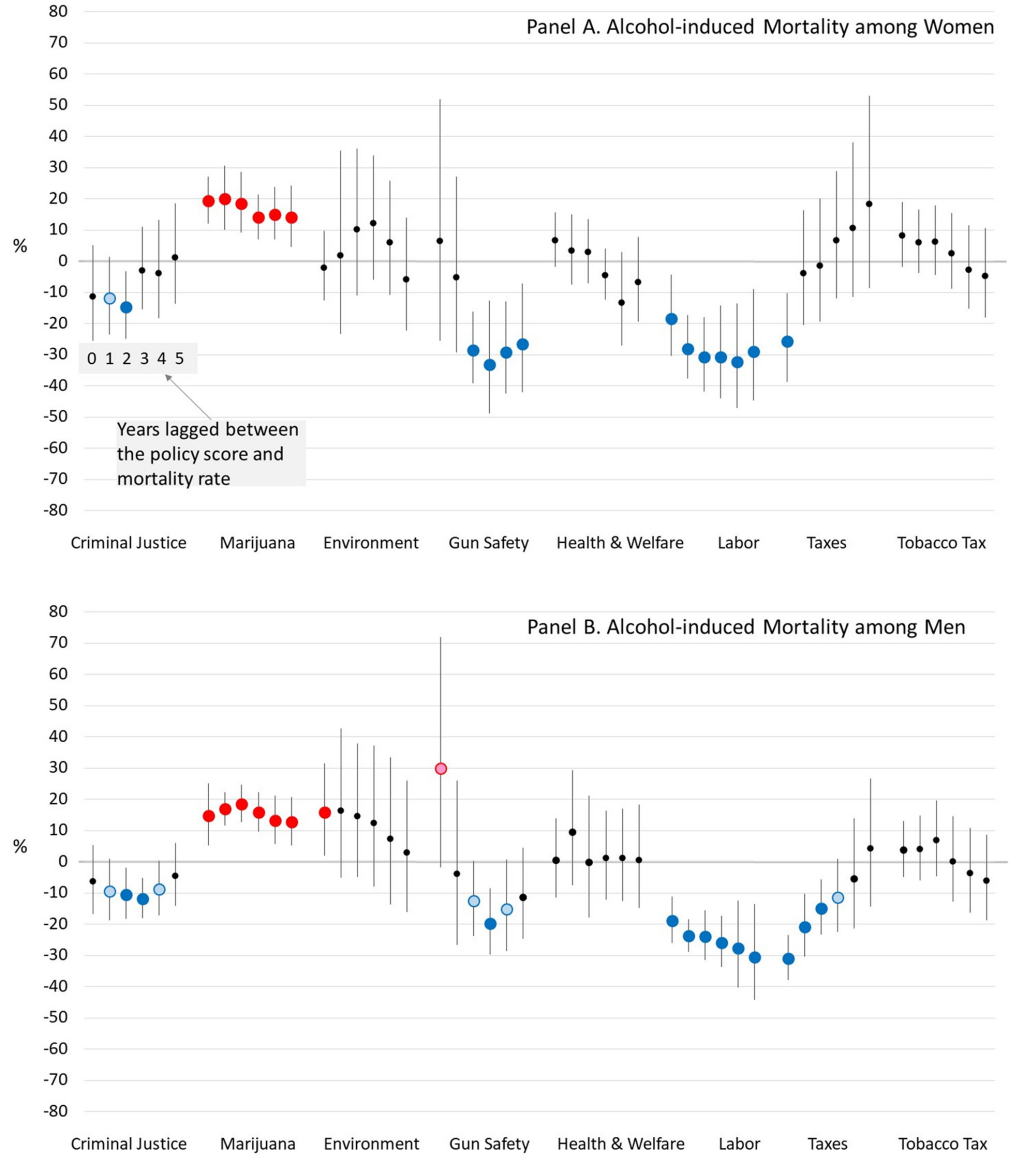
Estimated percent difference in alcohol-induced mortality rates when a U.S. State’s policy liberalism score is 1 versus 0, for various lag times between the policies and mortality. *Notes*: Blue dots mean a more liberal version of the policy is associated with lower mortality and red dots mean a more conservative version is associated with lower mortality. Dark blue and red dots indicate that the association is significant at *α*<0.05, while light blue and red dots indicate that it is significant at *α*<0.10.

Fig 4 demonstrates that, for women and men and across all lag times, lower working-age mortality from alcohol-induced causes was associated with more liberal labor policies and more conservative marijuana policies. An association between lower alcohol mortality and liberal gun safety policies was observed, but only for longer lag times. Among men, an association with more liberal criminal justice policies was observed for most lag times and with more liberal economic tax policy, but only for shorter lag times.

**Fig 5:**
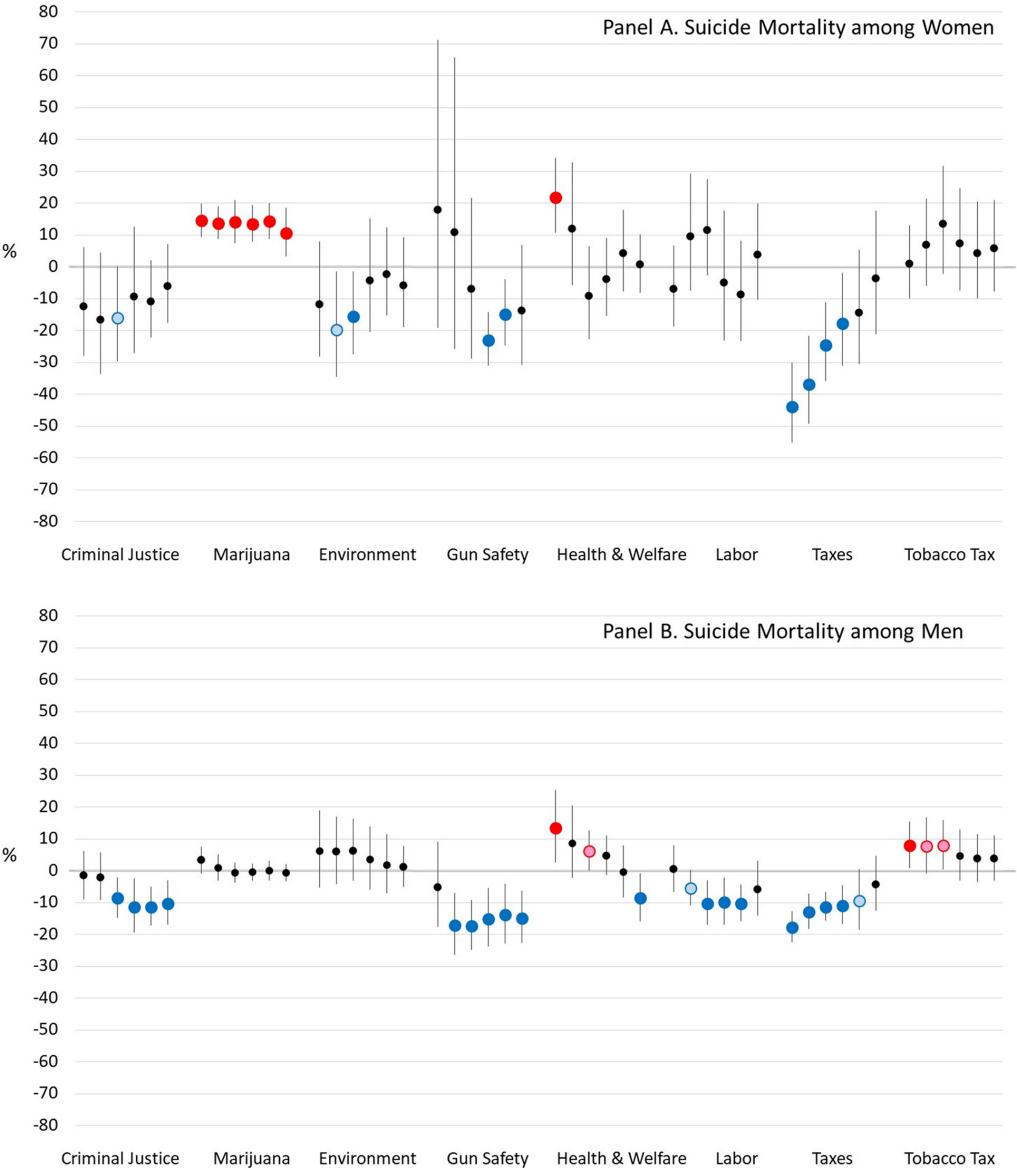
Estimated percent difference in suicide mortality rates when a U.S. State’s policy liberalism score is 1 versus 0, for various lag times between the policies and mortality. *Notes*: Blue dots mean a more liberal version of the policy is associated with lower mortality and red dots mean a more conservative version is associated with lower mortality. Dark blue and red dots indicate that the association is significant at *α*<0.05, while light blue and red dots indicate that it is significant at *α*<0.10.

Suicide rates among working-age adults were most closely associated with the marijuana and gun safety domains (Fig 5). Lower suicide rates among women were associated with more conservative marijuana policies, and among men (especially) and women, by more liberal gun safety policies. The strength of the association between suicide and other policy domains varied by sex and lag time. For instance, for most lag times, more liberal criminal justice and labor policy domains predicted lower suicide mortality among men. An association with economic tax policy was observed for suicide in men and women, but it waned with longer lag times.

**Fig 6:**
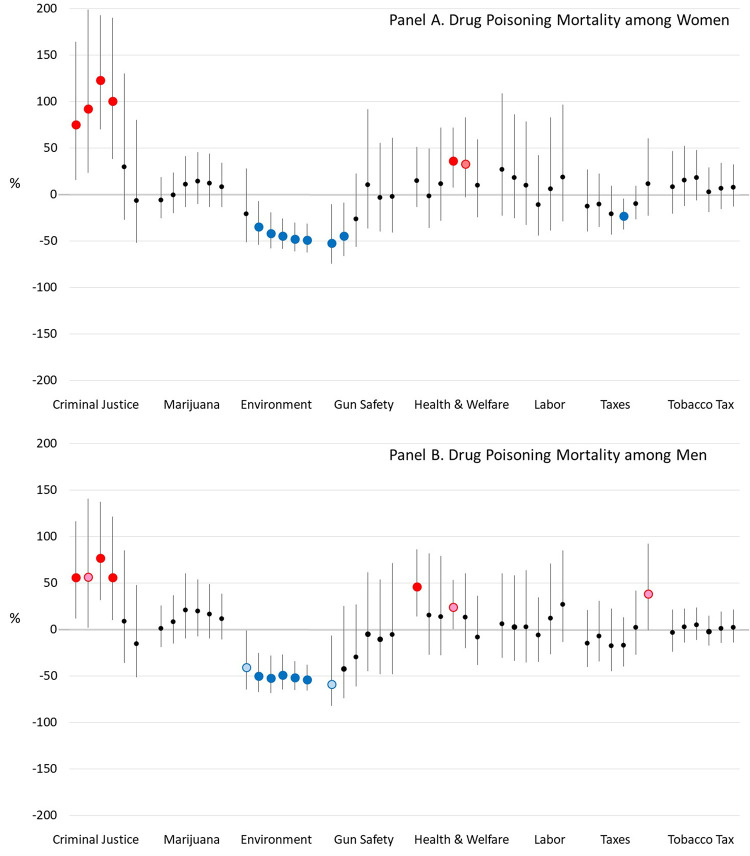
Estimated percent difference in drug poisoning mortality rates when a U.S. State’s policy liberalism score is 1 versus 0, for various lag times between the policies and mortality. *Notes*: Blue dots mean a more liberal version of the policy is associated with lower mortality and red dots mean a more conservative version is associated with lower mortality. Dark blue and red dots indicate that the association is significant at *α*<0.05, while light blue and red dots indicate that it is significant at *α*<0.10.

Fig 6 shows that drug poisoning mortality in midlife was associated with few of the eight policy domains, the most consistent being the association between more liberal environmental policies and lower drug poisoning mortality. An association between more conservative criminal justice policies and lower drug poisoning mortality weakened with longer lag times. We also note that the models showed that the annual number of states laws on opioid regulation and harm mitigation (which we included a time-varying covariate) was either associated with lower drug poisoning mortality in both women and men, or had no association, depending on the particular policy lag time. Table 2 summarizes the findings from all models.

**Table 2:** Summary of the strength and direction of the association between a policy domain’s liberalism score and working-age mortality rates.

	All-cause	CVD	Alcohol-induced	Suicide	Drug poisoning
Women					
Criminal justice					
Marijuana					
Environment					
Gun safety					
Health & welfare					
Labor					
Economic tax					
Tobacco tax					
Men					
Criminal justice					
Marijuana					
Environment					
Gun safety					
Health & welfare					
Labor					
Economic tax					
Tobacco tax					

*Notes*: The colors indicate the strength and direction of the association between the policy domains and mortality rates, as shown in Figs 2–6. The colors in Table 2 give most weight to the findings from models using lag times of either 3, 4, or 5 years between the policy score and the mortality rates. Dark blue means that a more liberal version of the policy domain was associated with lower mortality using all, or most of, these lag times; light blue indicates that a more liberal version was associated with lower mortality for some of these lag times, but the evidence was not as strong. Dark red and light red colors were assigned using the same logic, but for these policy domains a conservative version was associated with lower mortality rates.

### Hypothetical policy scenarios

We examined four counterfactual scenarios in which all policy domains in all states were set to the maximum liberal score of 1 (Scenario 1) or the maximum conservative score of 0 (Scenario 2); the maximum liberal score of 1 applied to all domains except marijuana and health and welfare, which were set to 0 and 0.5, respectively, because conservative marijuana policies were associated with lower all-cause mortality, and no association was observed for the health and welfare score (Scenario 3, “Hybrid”); and domains trending in conservative or liberal direction were set respectively to their 0 and 1 extremes (Scenario 4,”Status Quo”).

Estimates for each counterfactual scenario are in Table 3. In Scenario 1 (most liberal policies), the model predicted that the age-adjusted mortality rate among women ages 25–64 years would have been 168.6 deaths per 100,000 in 2019, a rate 33.7% lower than the actual age-adjusted rate (254.2 deaths per 100,000) that year. Among the 86,100,459 women in this age group in 2019, a 33.7% reduction in the actual *age-specific* mortality rates (i.e., multiplying them by 0.663) would have resulted in 86,181 fewer deaths. The predicted reduction in male mortality rates under the liberal scenario was 84,849 fewer deaths among men in this age group. Predicted mortality rates in Scenario 2 (most conservative policies) would have resulted in an estimated 217,635 more deaths (108,562 among women, 109,073 among men) than occurred in 2019. The hybrid scenario predicted the optimal results, saving an estimated 201,450 lives, whereas the status quo scenario predicted unfavorable results, an extra 69,210 deaths.

**Table 3:** Estimated change in the number of deaths in 2019 among adults ages 25–64 for hypothetical policy scenarios.

	Women			Men		
Policy Scenario	Simulated Age-adjusted Mortality Rate	Change from Actual Age-adjusted Rate	Change in Number of Deaths	Simulated Age-adjusted Mortality Rate	Change from Actual Age-adjusted Rate	Change in Number of Deaths
1. Maximum Liberal	168.6	-33.7%	-86,181	351.4	-20.1%	-84,849
2. Maximum Conservative	362.0	42.4%	108,562	553.7	25.9%	109,073
3. Hybrid	162.8	-36.0%	-92,057	325.7	-25.9%	-109,393
4. Status Quo	278.7	9.6%	24,643	486.4	10.6%	44,567

Notes: The liberal scenario set all policy scores to 1; the conservative scenario set all policy scores to 0; the hybrid scenario set all policy scores to 1 except for health and welfare, which was set to 0.5, and marijuana which was set to 0; the status quo scenario replaced scores for policy domains trending in a liberal or conservative direction on a national basis with 1 or 0, respectively. In 2019 there were 255,935 deaths among 86,100,459 women aged 25–64 and 421,559 deaths among 84,822,445 men aged 25–64 years (Source: CDC Wonder). Detailed calculations are available in S1 and S2 Tables in S1 File.

### Supplementary analyses

We conducted additional analyses to assess the robustness of our estimates. First, we examined how closely our models predict the actual age-adjusted mortality rates in 2019 by creating a simple average score for each policy domain in 2014 across the 50 states and then predicting the age-adjusted mortality rate in 2019 from the averages. The predicted rate (276 deaths per 100,000) differed from actual rate (254 deaths per 100,000) by just 5.1%, suggesting that the large counterfactual estimates for the above scenarios are not biased by a poorly predictive model.

We also estimated models for each of the eight policy domains, excluding the other seven domains, to explore whether collinearity among the domains shaped our findings. We compared the coefficient for each separately modeled policy domain to the coefficient for each domain estimated from a model that included the other seven domains. The results of these models, which are presented in S3–S5 Tables in S1 File, show little evidence of collinearity problems. The direction of the coefficients (positive or negative associations) was the same in both the unadjusted and adjusted models. As would be expected, the coefficients tended to be marginally smaller in the adjusted model. One exception is the health and welfare domain, an important predictor of women’s all-cause mortality in the unadjusted model, but the coefficient diminished in size and significance after adjustment for the other policy domains. We infer from this pattern that the health and welfare domain does not provide much new information for predicting women’s working-age mortality rates, net of the other seven policy domains.

The supplement also provides bivariate correlations among the domains as well as Variance Inflation Factors (VIF) for each domain (S6 and S7 Tables in S1 File, respectively). These analyses also indicate that collinearity does not adversely impact our findings. The VIFs range from 1.2 to 2.7, below common thresholds (VIF>10 or VIF>4) for signaling problematic collinearity [39].

Lastly, we re-estimated the all-cause mortality models, while adjusting for the remainder of the policy domains created by Grumbach (abortion, campaign finance, civil rights, education, housing and transportation, immigration, LGBT, public labor, voting) which did not meet our inclusion criteria. This demonstrated that our 8-domain model was robust to the inclusion of all other domains (S8 Table in S1 File): in both the 8-domain model and all-domain model, the coefficients for the eight domains of ft had the same direction and similar magnitude and their statistical significance was unchanged. One exception was the economic tax coefficient for women: although it was no longer statistically significant when all domains were included in the model, 95% confidence intervals for the coefficient in the 8-domain model overlapped with the interval from the all-domain model.

## Discussion

On average, Americans die younger than their peers in most other high-income countries. In a 2013 U.S. survey, 85% of adult respondents indicated that their ideal life span was 79 years or older, yet U.S. life tables predicted that only 60% of people born that year could expect to survive to age 79 [40, 41]. Our findings, which examine working-age deaths among adults ages 25–64 years, suggest state policies–specifically, their left/right lean–may be a contributing factor and provide new insights into potential strategies to reduce working-age mortality.

Three findings from this associational study are particularly intriguing. First, during the 1999 to 2019 period, statistically significant associations existed between the all-cause mortality rates of working-age adults and the policies of their state of residence. The association extended to seven of the eight policy domains, net of each other (the exception being the health and welfare policy domain). Lower mortality rates among working-age men and women were predicted by more liberal policies on the environment, gun safety, labor, economic taxes, and tobacco taxes in their state of residence, and by more conservative policies on marijuana. These associations were robust across all lag times examined. In addition, a more liberal criminal justice policy predicted lower all-cause mortality rates, but the association was comparatively weak and only apparent after a 3-year lag time. These findings are generally consistent with prior research. Specifically, a previous study found the same domains, except criminal justice, to be predictive of changes in state-level life expectancy between 1970 and 2014 [20].

Second, each policy domain was associated with some causes of death but not others, which may provide clues about potential pathways through which the policy domains shape working-age mortality. Where we had strong expectations based on prior research, the associations generally supported them, providing added assurance about the robustness of our findings. For example, the tobacco tax domain was related to deaths from CVD, but not to deaths from alcohol-induced causes, suicide, or drug poisonings. Also supporting expectations, the gun safety domain was robustly associated with suicide mortality among men and less so among women.

However, some policy domains may be associated with specific causes of death through more indirect pathways. For instance, more liberal economic tax policies were strongly associated with lower CVD mortality rates. Tax policies like earned income tax credits and income taxes can enhance socioeconomic conditions, an important predictor of CVD and cardiac risk factors (e.g., smoking, nutrition) [42–44]. A prior comprehensive analysis showed that states’ tax policies predict economic wellbeing and a host of health outcomes, from health behaviors to mortality [45].

More liberal labor policies were strongly associated with lower alcohol-induced mortality among women and men. Favorable macroeconomic conditions, which can be associated with labor policies, appear to lower the risk of alcohol consumption and problem drinking [46] and suicide [47]. The labor domain includes policies such as minimum wage and paid leave, which can reduce financial and emotional stress, smoking, obesity, and teenage pregnancy, as well as improve medical care access, housing, nutrition, and exercise [48–52]. Whether these or other policies explain the association with alcohol-induced disease requires further research.

A few policy domains may have complex associations with specific causes of death. For example, although the association between gun safety policies and suicides is intuitive—such policies are associated with lower rates of homicide, suicide, and firearm-related deaths [53]—the association with CVD and deaths from alcohol-induced causes is less clear. Gun safety policies may have broader and more diffuse effects on population health and mortality. For instance, each day, approximately 235 nonfatal firearm injuries occur in the United States [54]. A study of the 2009–2017 period found that, on average, each year there were an astonishing 85,894 nonfatal firearm injuries [54]. Nonfatal injuries can cause a host of life-threatening issues such as physical impairment, disability, morbidity, and poor mental health [55]. More broadly, gun violence has ripple effects, including not only the nonfatal and fatal physical injuries that result from the shootings themselves but also indirect health effects resulting in long-term disabilities, psychosocial trauma and stress, incarceration and its health consequences, and the adverse health effects of economic instability among affected families and communities, among others. The unhealthy and complex ecosystem surrounding gun violence could increase the risk of multiple diseases, explaining the observed association with deaths from CVD, alcohol-induced causes, and other causes not examined here.

Similarly, the relationship between environment policies and drug poisoning deaths could have complex explanations. Changes in environmental policies during the period may correlate with changes in unmeasured but pertinent state-level characteristics. For instance, states with more lax environmental policies tend to have high shares of extractive and construction industries, where workers bear a high risk of drug use and overdose [56, 57]. If changes in states’ environmental policies in recent decades correspond with changes in these industries and occupations, this may account for the association that we found between environmental policies and working-age mortality. Further investigation of these complex associations and their potential pathways is needed.

Two recent comprehensive reviews found inconclusive evidence on the health impacts of states’ marijuana policies and of marijuana use [58, 59]. A National Academies of Sciences, Engineering, and Medicine review [58] identified some benefits of marijuana use, such as effectively treating chronic pain, but also potential harms, such as increased risk of developing alcohol dependence and/or abuse, depressive disorders, and schizophrenia, as well as higher risks of motor vehicle crashes and suicide ideation, attempts, and completion. Studies examining the impact of states’ marijuana legalization on opioid-related mortality have also produced mixed findings [60–63]. Rapidly evolving and heterogenous marijuana policies, plus potentially different consequences for individuals (e.g., pain relief) versus populations (e.g., externalities such as motor vehicle crashes) make it challenging to reconcile the disparate results. Our finding that more conservative marijuana policies were associated with lower alcohol-induced mortality and suicide (for women) is compatible with several of the studies mentioned above. However, this association should be interpreted cautiously, bearing in mind that it does not establish causality and that the overall evidence on the health impact of marijuana policies and use is mixed.

Our third finding is that changing the liberal-conservative orientation of the policy domains within a state predicts meaningful differences in that state’s working-age mortality rates. As an example, the difference in mortality rates associated with gun safety policies with a maximum liberal versus maximum conservative score would be an estimated 20.6% among working-age men, net of the other policy domains. The estimated impact of changing policies across multiple domains is dramatic. Our simulations estimate that 171,030 lives would have been saved in 2019 if all states had a maximum liberal orientation in all eight policy domains. Conversely, our simulation predicts that an additional 217,635 working-age deaths would have occurred in 2019—the equivalent of a 600-passenger airplane crashing every day of the year—if all states had a maximum conservative orientation. These deaths involve adults when they are in the prime of their lives. Although it is unlikely that all states’ policy contexts would move to either end on the political spectrum, simply moving towards the extremes could potentially have staggering consequences. Lastly, the largest number of predicted lives saved (201,450) came from a hybrid scenario, with maximum conservative marijuana policies and maximum liberal policies on labor, environment, gun safety, criminal justice, economic taxes, and tobacco taxes.

These state policies could adversely affect health and mortality for all age groups but are particularly salient for working-age adults, which may help explain the high and rising mortality among this age group and, more broadly, the stagnation in U.S. life expectancy. For example, the labor domain includes policies such as paid sick leave, right to work laws, disability insurance, and minimum wage levels, all of which are particularly salient for working-age adults. The economic tax domain, which includes policies such as income tax levels and state supplemental earned income tax credits that are targeted to working families with children, is also relevant for working-age adults [64]. Policy domains unrelated to employment and economics, such as criminal justice, are also salient. In 2020, adults ages 25–64 years represented 81% of adult inmates in local jails [65] and 88% of adult prisoners under federal or state jurisdiction [66]. State tobacco taxes have a stronger impact on smoking prevalence among adults 25–64 (especially those ages 18–24) than those 65 and older [25], and adults 25–64 are considerably more likely to smoke (approximately 14.5%) than are adults 65 and older (9.0%) and adults ages 18–24 (7.4%) [67]. Lastly, firearm safety policies may also be particularly relevant for working-age adults given their relatively high levels of gun ownership. Adults ages 30 and younger are the least likely to own guns, while working-age adults are either more or similarly likely to own guns as older adults, depending on the study [68–70].

Trends in the share of the working-age population residing in states with conservative-leaning or liberal-leaning policies provide additional context for our findings. In supplementary analyses (S9 Table in S1 File), we estimated the share of the working-age population in 1994 and 2014 residing in states with relatively conservative policies (scoring below the 21-year average) on each of the eight policy domains. As an example, the average score for the economic tax policy domain across the 1994–2014 period was 0.53. The share of working-age adults residing in states scoring below 0.53 fell slightly, from 43.6% in 1994 to 38.2% in 2014. This may have benefitted mortality given the association between conservative economic tax policy and higher working-age mortality estimated from our analyses. We also observed a smaller share of working-age adults living in states with below average (i.e., more conservative) scores on the criminal justice, environment, and tobacco tax domains. In sharp contrast, the percentage of working-age adults living in states with relatively conservative firearm policies nearly doubled, from 28.9% in 1994 to 54.6% in 2014; the percentage living in states with relatively conservative labor policies increased considerably, from 37.4% to 51.4%; and the percentage living in states with relatively conservative marijuana policies declined considerably, from 67.0% to 44.5%. That is, a growing share of working-age adults are being exposed to these three policy changes that are associated with elevated mortality.

The emergence of more conservative state policies across several domains and shifts in the share of the population living in states governed by these policies represent only partial explanations for the high and rising mortality among working-age Americans and the overall mortality disadvantage of the United States compared to other high-income countries. As noted in the introduction, the health and longevity of Americans is shaped by a web of layered influences at the individual, meso, and macro levels, and the latter encompasses not only state policies but also factors such as national policies, corporations, and social values.

### Limitations and directions for future research

Strengths of the study include the use of robust statistical methods to examine 21 years of mortality data and a large number of domains spanning more than 100 policies, but we are careful to note several limitations. First, the study reports associations and therefore cannot establish whether the policy domains have a causal relationship mortality, although the findings generally concur with causal evidence from studies that isolated the effects of specific policies [e.g., 43, 50–52, 71–73]. Second, our findings may not apply outside of working ages.

Third, the analyses did not include all state policies, so our findings may represent a lower bound on their potential contribution to working-age mortality rates. Fourth, as noted earlier, because the model included fixed effects for states, it does not include state characteristics that were relatively constant across states during the study period, such as religious orientation and racial composition. Fifth, although we included the time-varying share of immigrants and unemployment rates by state, confounding by unmeasured factors that varied over time cannot be ruled out. That is, some of the policy domains examined in this study could have changed in step with unmeasured policies or characteristics that are relevant for mortality. Sixth, we did not include traditional social determinants, such as education levels or income, or health behaviors, which in this context are conceptualized as pathways or mediators through which policies might affect mortality; controlling for pathways would bias the associations between the policy domains and mortality [74, 75]. Relatedly, we note that states with populations whose human capital is rising (e.g., through educational attainment) may see health gains. Such changes are likely part of the explanation for diverging state-level mortality trends since the mid-1980s; however, available evidence shows that they have played only a small role [32, 76]. In an recent analysis, Couillard and colleagues found that changes in education and income levels within states’ populations played a negligible role in the divergence in working-age mortality rates between states [32]. They concluded that the divergence reflected the differential adoption of policies like tobacco taxes, Medicaid expansion, and income support in certain states [32].

Lastly, interstate migration could potentially influence the results. For example, states gaining immigrant populations or interstate migrants may have seen population health increase because of a healthy (im)migrant effect. Studies that have examined the potential role of immigration on state-level mortality trends in recent decades do not find a particularly strong role [76, 77]. Similarly, although using a more granular unit of analysis, Ezzati and colleagues concluded that the growing divide in mortality rates across counties was unlikely due to different patterns of (im)migration [78]. In sum, while we do not rule out migration effects, available evidence indicates that any such effects would not materially alter our findings.

Priorities for future research include moving beyond demonstrations of associations to understand causal pathways; investigating how “bundles” of policies shape mortality, given that states have increasingly implemented sets of either left-leaning or right-leaning policies across multiple policy domains [21, 79]; examining whether the impact of states’ policy contexts on mortality differs by race, education, urban-rural settings, and other population subgroups; and exploring the degree to which policy domains have synergistic or countervailing effects on mortality. It may also be informative to explore the extent to which the health effects of state policy contexts are influenced by national policy context, such as in state Medicaid expansion under provisions of the federal Affordable Care Act.

## Conclusions

The large and growing mortality disadvantage of working-age U.S. adults may partly reflect changes in state policy contexts that have occurred in recent decades. Policies that promote gun safety, environmental protections, labor (e.g., minimum wage, paid leave), progressive taxation, and tobacco control are among the potentially important policy opportunities to address increasing working-age mortality at the macrostructural level. The health gains from such policies offer potential collateral benefits to families, the economy, workers, and the health care system. More studies are needed assess how the increasing bundling of state policies into either left-leaning or right-leaning has affected the divergence in population health and mortality across U.S. states.

## Supporting information

S1 File Supplementary analyses. (DOCX)
