# Supplementary material for: U.S. state policy contexts and mortality of working-age adults
Source: PLoS One. 2022 Oct 26;17(10):e0275466. doi: 10.1371/journal.pone.0275466 (PMC9604945; doi:10.1371/journal.pone.0275466)
Supplement: S1 File — (DOCX) [file pone.0275466.s001.docx]

**S1 Fig 1.** **Trends in state policy domains for each U.S. state, 1995-2014**


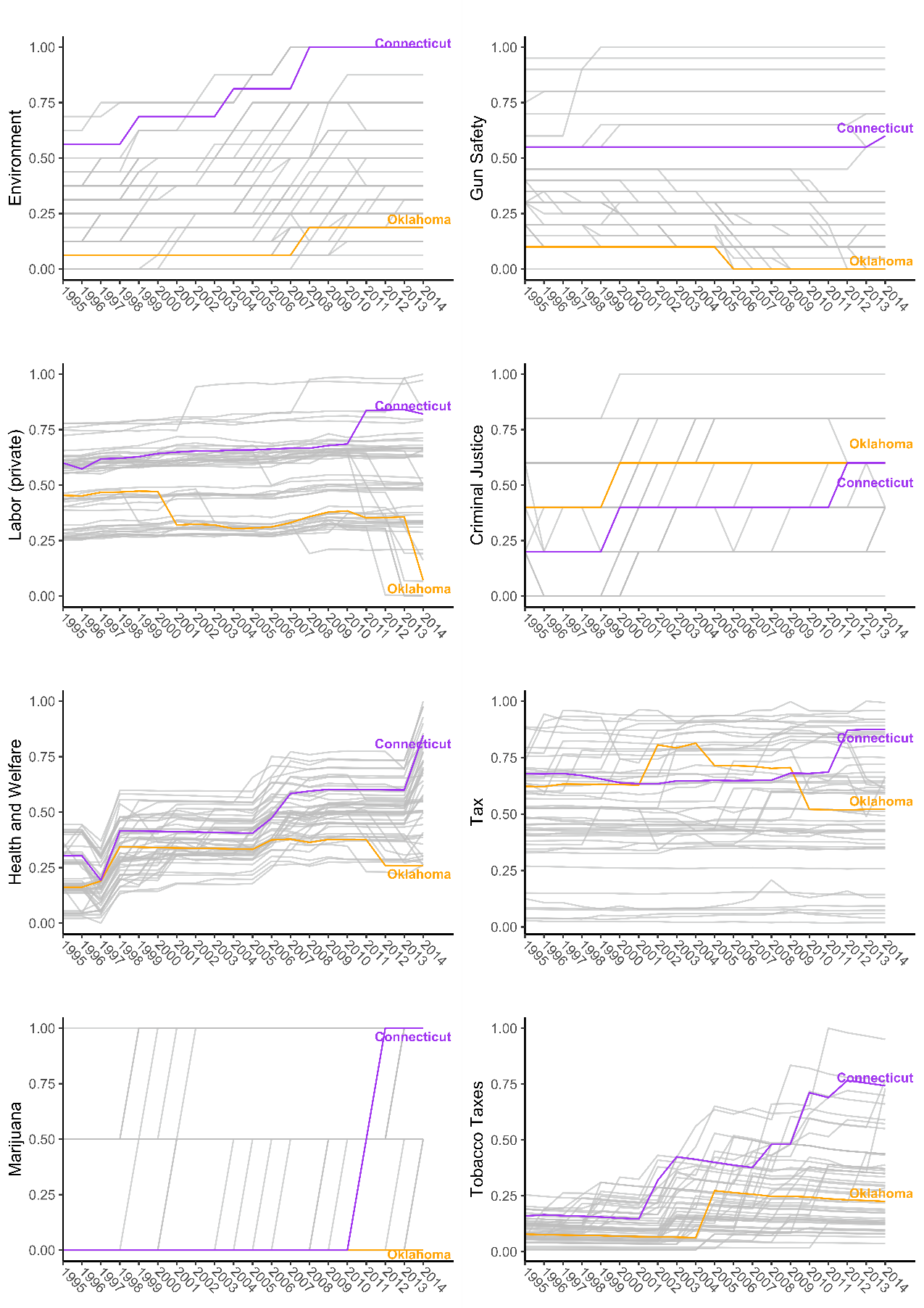


*Note:* each panel contains a line for every state, showing how the state’s policy liberalism score trended during the study period (scores range from 0 to 1 on a conservative to liberal continuum)

| **S1 Table 1. Actual and estimated deaths and mortality rates among women ages 25–64** | | | | | | | | | |
| --- | --- | --- | --- | --- | --- | --- | --- | --- | --- |
|  |  | | |  | Estimated Deaths in 2019 for Hypothetical Policy Scenarios | | | | |
|  | Population, Deaths, and Age-standardized Mortality Rates (ASMR) in 2019 | | |  | Maximum Liberal | Maximum Conservative | Hybrid | Current Policy Direction | Average Policy Context in 2014 |
| Age Group | Population | Deaths | ASMR |  | ASMR*0.663 | ASMR*1.424 | ASMR*0.640 | ASMR*1.096 | ASMR*1.085 |
| 25-34 | 22,581,141 | 17,827 | 0.000789 |  | 0.000524 | 0.001124 | 0.000506 | 0.000865 | 0.000856 |
| 35-44 | 20,867,064 | 29,550 | 0.001416 |  | 0.000939 | 0.002017 | 0.000907 | 0.001552 | 0.001536 |
| 45-54 | 20,702,936 | 61,546 | 0.002973 |  | 0.001972 | 0.004234 | 0.001904 | 0.003259 | 0.003224 |
| 55-64 | 21,949,318 | 147,012 | 0.006698 |  | 0.004442 | 0.009539 | 0.004289 | 0.007343 | 0.007264 |
| Total | 86,100,459 | 255,935 |  |  |  |  |  |  |  |
|  |  |  |  |  |  |  |  |  |  |
| *Estimated number of deaths in 2019 for each scenario:^a^* | | | |  | *169,754* | *364,497* | *163,878* | *280,578* | *277,565* |
| *Estimated deaths for each scenario – actual deaths in 2019* | | | |  | *-86,181* | *108,562* | *-92,057* | *24,643* | *21,630* |

^a^ Obtained by multiplying the scenario’s mortality rate for each age group by the population size of that group, and then summing the results.

| **S1 Table 2. Actual and estimated deaths and mortality rates among men ages 25–64** | | | | | | | | | |
| --- | --- | --- | --- | --- | --- | --- | --- | --- | --- |
|  |  | | |  | Estimated Deaths in 2019 for Hypothetical Policy Scenarios | | | | |
|  | Population, Deaths, and Age-standardized Mortality Rates (ASMR) in 2019 | | |  | Maximum Liberal | Maximum Conservative | Hybrid | Current Policy Direction | Average Policy Context in 2014 |
| Age Group | Population | Deaths | ASMR |  | ASMR*0.799 | ASMR*1.259 | ASMR*0.741 | ASMR*1.106 | ASMR*1.066 |
| 25-34 | 23,359,180 | 41,351 | 0.0017702 |  | 0.0014139 | 0.0022282 | 0.0013109 | 0.0019574 | 0.0018864 |
| 35-44 | 20,792,080 | 53,436 | 0.0025700 |  | 0.0020527 | 0.0032350 | 0.0019031 | 0.0028417 | 0.0027387 |
| 45-54 | 20,171,966 | 98,847 | 0.0049002 |  | 0.0039139 | 0.0061681 | 0.0036286 | 0.0054183 | 0.0052219 |
| 55-64 | 20,499,219 | 227,925 | 0.0111187 |  | 0.0088808 | 0.0139955 | 0.0082334 | 0.0122942 | 0.0118486 |
| Total | 84,822,445 | 421,559 |  |  |  |  |  |  |  |
|  |  |  |  |  |  |  |  |  |  |
| *Estimated number of deaths in 2019 for each scenario:^a^* | | | |  | *336,710* | *530,632* | *312,166* | *466,126* | *449,232* |
| *Estimated deaths for each scenario – actual deaths in 2019:* | | | |  | *-84,849* | *109,073* | *-109,393* | *44,567* | *27,673* |

^a^ Obtained by multiplying the scenario’s mortality rate for each age group by the population size of that group, and then summing the results.

| **S1 Table 3. Regression coefficients predicting log(all-cause mortality rate) from U.S. state policies, adults ages 25-64 years, 1999-2019** | | | | | |
| --- | --- | --- | --- | --- | --- |
|  | Women | |  | Men | |
|  | Model 1^b^ | Model 2 |  | Model 1^b^ | Model 2 |
| Time (1999-2019) | -0.002 (0.001)† | -0.001 (0.001) |  | -0.002 (0.001)* | -0.001 (0.001) |
| State policy domains ^a^ |  |  |  |  |  |
| Environment | -0.124 (0.021)*** | -0.052 (0.009)*** |  | -0.081 (0.012)*** | -0.035 (0.010)** |
| Gun safety | -0.228 (0.017)*** | -0.166 (0.013)*** |  | -0.143 (0.013)*** | -0.100 (0.012)*** |
| Labor (private) | -0.111 (0.023)*** | -0.039 (0.013)** |  | -0.076 (0.017)*** | -0.033 (0.012)* |
| Criminal justice | -0.032 (0.014)* | -0.025 (0.009)* |  | -0.027 (0.011)* | -0.022 (0.009)* |
| Health and welfare | -0.043 (0.011)** | -0.005 (0.012) |  | -0.027 (0.017) | -0.004 (0.017) |
| Economic tax | -0.049 (0.014)** | -0.024 (0.008)** |  | -0.023 (0.009)* | -0.013 (0.009) |
| Marijuana | 0.011 (0.004)** | 0.018 (0.003)*** |  | 0.030 (0.004)*** | 0.035 (0.005)*** |
| Tobacco taxes | -0.059 (0.014)*** | -0.040 (0.004)*** |  | -0.037 (0.010)** | -0.026 (0.004)*** |
| Covariates |  |  |  |  |  |
| Sum of opioid policies | - | 0.004 (0.002)† |  | - | 0.004 (0.002)† |
| Percent immigrants | - | -0.004 (0.002)† |  | - | -0.005 (0.002)* |
| Percent unemployed | - | -0.003 (0.001)*** |  | - | -0.004 (0.001)*** |
| Constant | - | 2.609 (0.026)*** |  | - | 2.812 (0.027)*** |
|  |  |  |  |  |  |
| N | 1,050 | 1,050 |  | 1,050 | 1,050 |
| F-statistic | - | 3,404*** |  | - | 2,833*** |
| R2 (within) | - | 0.591 |  | - | 0.552 |

****p*<0.001; ***p*<0.01; **p*<0.05; †*p*<0.10

*Notes:* All models include fixed effects for states and, for each state-year observation, the sum of opioid policies, the percentage of the state’s population that are immigrants, and the unemployment percentage.

^a^ Each domain is scaled from 0 to 1, where 0 is maximum conservative and 1 is maximum liberal observed.

^b^ Coefficients from eight separate models examining one policy domain at a time.

| **S1 Table 4.** **Regression coefficients predicting log(cause-specific mortality rate) from U.S. state policies, women ages 25-64 years, 1999-2019** | | | | | | | | |
| --- | --- | --- | --- | --- | --- | --- | --- | --- |
|  | Cardiovascular | | Alcohol Induced | | Suicide | | Drug Poisoning | |
|  | Model 1^b^ | Model 2 | Model 1^b^ | Model 2 | Model 1^b^ | Model 2 | Model 1^b^ | Model 2 |
| Time (1999-2019) | -0.007 (0.001)*** | -0.005 (0.001)*** | 0.015 (0.001)*** | 0.015 (0.001)*** | 0.011 (0.001)*** | 0.011 (0.001)*** | 0.022 (0.007)** | 0.024 (0.009)* |
| State policy domains ^a^ |  |  |  |  |  |  |  |  |
| Environment | -0.164 (0.024)*** | -0.089 (0.014)*** | -0.118 (0.043)* | -0.026 (0.042) | -0.035 (0.034) | -0.026 (0.033) | -0.251 (0.067)** | -0.291 (0.065)*** |
| Gun safety | -0.261 (0.024)*** | -0.177 (0.021)*** | -0.210 (0.065)** | -0.134 (0.052)* | -0.062 (0.043) | -0.065 (0.048) | -0.110 (0.131) | -0.010 (0.111) |
| Labor (private) | -0.124 (0.028)*** | -0.025 (0.023) | -0.184 (0.044)*** | -0.149 (0.055)* | -0.019 (0.038) | 0.016 (0.032) | -0.066 (0.100) | 0.075 (0.112) |
| Criminal justice | -0.052 (0.018)** | -0.043 (0.016)* | -0.011 (0.035) | 0.005 (0.035) | -0.030 (0.030) | -0.027 (0.029) | -0.035 (0.159) | -0.028 (0.145) |
| Health and welfare | -0.059 (0.018)** | -0.012 (0.018) | -0.058 (0.028)* | -0.030 (0.032) | -0.004 (0.022) | 0.003 (0.020) | 0.019 (0.102) | 0.042 (0.082) |
| Economic tax | -0.076 (0.020)** | -0.039 (0.012)** | 0.046 (0.059) | 0.073 (0.057) | -0.009 (0.039) | -0.016 (0.044) | 0.034 (0.067) | 0.047 (0.081) |
| Marijuana | -0.013 (0.004)* | -0.003 (0.005) | 0.055 (0.021)* | 0.057 (0.019)** | 0.042 (0.015)* | 0.044 (0.015)** | 0.031 (0.053) | 0.034 (0.048) |
| Tobacco taxes | -0.071 (0.015)*** | -0.047 (0.011)*** | -0.032 (0.048) | -0.021 (0.033) | 0.016 (0.030) | 0.024 (0.030) | 0.013 (0.042) | 0.033 (0.046) |
| Covariates |  |  |  |  |  |  |  |  |
| Sum of opioid policies | - | 0.008 (0.002)** | - | 0.011 (0.004)* | - | 0.011 (0.003)** | - | 0.002 (0.017) |
| Percent immigrants | - | -0.002 (0.002) | - | -0.016 (0.007)* | - | -0.007 (0.004)† | - | 0.027 (0.015)† |
| Percent unemployed | - | -0.005 (0.001)*** | - | -0.004 (0.002)* | - | -0.001 (0.001) | - | 0.010 (0.007) |
| Constant | - | 1.985 (0.023)*** | - | 0.940 (0.065)*** | - | 0.675 (0.054)*** | - | 0.504 (0.194)* |
|  |  |  |  |  |  |  |  |  |
| N | 1,050 | 1,050 | 1,050 | 1,050 | 1,050 | 1,050 | 1,050 | 1,050 |
| F-statistic | - | 367.6*** | - | 1,911*** | - | 2,803*** | - | 183.9*** |
| R2 (within) | - | 0.779 | - | 0.709 | - | 0.670 | - | 0.722 |

**p*<0.001; ***p*<0.01; **p*<0.05; †*p*<0.10

*Notes:* All models include fixed effects for states and, for each state-year observation, the sum of opioid policies, the percentage of the state’s population that are immigrants, and the unemployment percentage.

^a^ Each domain is scaled from 0 to 1, where 0 is maximum conservative and 1 is maximum liberal observed.

^b^ Coefficients from eight separate models examining one policy domain at a time.

| **S1 Table 5.** **Regression coefficients predicting log(cause-specific mortality rate) from U.S. state policies, men ages 25-64 years, 1999-2019** | | | | | | | | |
| --- | --- | --- | --- | --- | --- | --- | --- | --- |
|  | Cardiovascular | | Alcohol Induced | | Suicide | | Drug Poisoning | |
|  | Model 1^b^ | Model 2 | Model 1^b^ | Model 2 | Model 1^b^ | Model 2 | Model 1^b^ | Model 2 |
| Time (1999-2019) | -0.006 (0.001)*** | -0.005 (0.001)*** | 0.007 (0.001)*** | 0.007 (0.001)*** | 0.008 (0.001)*** | 0.009 (0.000)*** | 0.021 (0.005)** | 0.025 (0.007)*** |
| State policy domains ^a^ |  |  |  |  |  |  |  |  |
| Environment | -0.078 (0.012)*** | -0.036 (0.010)** | -0.062 (0.042) | 0.012 (0.045) | -0.033 (0.021) | 0.005 (0.014) | -0.295 (0.084)** | -0.335 (0.065)*** |
| Gun safety | -0.144 (0.016)*** | -0.103 (0.016)*** | -0.112 (0.045)* | -0.052 (0.036) | -0.083 (0.025)** | -0.070 (0.021)** | -0.156 (0.153) | -0.023 (0.131) |
| Labor (private) | -0.055 (0.014)*** | -0.005 (0.015) | -0.157 (0.029)*** | -0.158 (0.048)** | -0.059 (0.024)* | -0.026 (0.020) | -0.061 (0.085) | 0.103 (0.084) |
| Criminal justice | -0.017 (0.015) | -0.013 (0.013) | -0.031 (0.024) | -0.020 (0.023) | -0.056 (0.020)* | -0.047 (0.017)* | -0.082 (0.134) | -0.071 (0.123) |
| Health and welfare | -0.039 (0.016)* | -0.015 (0.016) | -0.017 (0.032) | 0.002 (0.036) | -0.053 (0.014)** | -0.039 (0.018)* | -0.065 (0.100) | -0.036 (0.087) |
| Economic tax | -0.049 (0.013)*** | -0.033 (0.007)*** | 0.002 (0.046) | 0.018 (0.043) | -0.036 (0.017)* | -0.019 (0.020) | 0.122 (0.047)* | 0.141 (0.073)† |
| Marijuana | -0.003 (0.006) | 0.003 (0.006) | 0.049 (0.018)* | 0.052 (0.015)** | -0.007 (0.005) | -0.003 (0.006) | 0.048 (0.052) | 0.048 (0.048) |
| Tobacco taxes | -0.047 (0.011)*** | -0.033 (0.008)*** | -0.024 (0.043) | -0.027 (0.032) | 0.009 (0.021) | 0.016 (0.015) | -0.019 (0.035) | 0.010 (0.038) |
| Covariates |  |  |  |  |  |  |  |  |
| Sum of opioid policies | - | 0.007 (0.002)** | - | 0.008 (0.004)* | - | -0.001 (0.002) | - | 0.017 (0.014) |
| Percent immigrants | - | -0.005 (0.002)** | - | -0.011 (0.006)† | - | -0.005 (0.002)* | - | 0.015 (0.015) |
| Percent unemployed | - | -0.004 (0.001)*** | - | -0.004 (0.001)** | - | 0.001 (0.001) | - | -0.005 (0.005) |
| Constant | - | 2.289 (0.019)*** | - | 1.414 (0.065)*** | - | 1.407 (0.031)*** | - | 0.968 (0.195)*** |
|  |  |  |  |  |  |  |  |  |
| N | 1,050 | 1,050 | 1,050 | 1,050 | 1,050 | 1,050 | 1,050 | 1,050 |
| F-statistic | - | 816 | - | 913*** | - | 737*** | - | 371.7*** |
| R2 (within) | - | 0.833 | - | 0.492 | - | 0.712 | - | 0.743 |

**p*<0.001; ***p*<0.01; **p*<0.05; †*p*<0.10

*Notes:* All models include fixed effects for states and, for each state-year observation, the sum of opioid policies, the percentage of the state’s population that are immigrants, and the unemployment percentage.

^a^ Each domain is scaled from 0 to 1, where 0 is maximum conservative and 1 is maximum liberal observed.

^b^ Coefficients from eight separate models examining one policy domain at a time.

| **S1 Table 6. Pearson’s correlation coefficients among eight policy domains, using U.S. state-year observations for 1994–2014** | | | | | | | | |
| --- | --- | --- | --- | --- | --- | --- | --- | --- |
|  | Criminal justice | Economic tax | Environment | Firearm safety | Health & welfare | Marijuana | Private labor | Tobacco tax |
| Criminal justice | 1.00 |  |  |  |  |  |  |  |
| Economic tax | 0.09 | 1.00 |  |  |  |  |  |  |
| Environment | 0.06 | 0.46 | 1.00 |  |  |  |  |  |
| Firearm safety | 0.05 | 0.42 | 0.57 | 1.00 |  |  |  |  |
| Health & welfare | 0.30 | 0.29 | 0.54 | 0.27 | 1.00 |  |  |  |
| Marijuana | 0.04 | 0.16 | 0.35 | 0.11 | 0.26 | 1.00 |  |  |
| Private labor | 0.20 | 0.43 | 0.56 | 0.61 | 0.47 | 0.34 | 1.00 |  |
| Tobacco tax | 0.29 | 0.27 | 0.65 | 0.35 | 0.63 | 0.35 | 0.49 | 1.00 |

| **S1 Table 7. Variance inflation factors for the eight policy domains** | |
| --- | --- |
| Policy domain | Variance Inflation Factor |
| Criminal justice | 1.2 |
| Economic tax | 1.4 |
| Environment | 2.7 |
| Firearm safety | 2.0 |
| Health & welfare | 1.9 |
| Marijuana | 1.3 |
| Private labor | 2.2 |
| Tobacco tax | 2.3 |

| **S1 Table 8. Regression coefficients predicting log(all-cause mortality rate) from U.S. state policies, with and without adjusting for other policy domains in Grumbach (2018), adults ages 25-64 years, 1999-2019** | | | | | |
| --- | --- | --- | --- | --- | --- |
|  | Women | |  | Men | |
|  | Model not adjusted for other domains | Model adjusted for other domains^b^ |  | Model not adjusted for other domains | Model adjusted for other domains^b^ |
| Time (1999-2019) | -0.001 (0.001) | 0.000 (0.001) |  | -0.001 (0.001) | 0.000 (0.001) |
| State policy domains^a^ |  |  |  |  |  |
| Environment | -0.052 (0.009)*** | -0.063 (0.010)*** |  | -0.035 (0.010)** | -0.049 (0.011)*** |
| Gun safety | -0.166 (0.013)*** | -0.166 (0.018)*** |  | -0.100 (0.012)*** | -0.118 (0.017)*** |
| Labor (private) | -0.039 (0.013)** | -0.047 (0.013)** |  | -0.033 (0.012)* | -0.045 (0.015)** |
| Criminal justice | -0.025 (0.009)* | -0.023 (0.009)* |  | -0.022 (0.009)* | -0.022 (0.008)** |
| Health and welfare | -0.005 (0.012) | 0.000 (0.012) |  | -0.004 (0.017) | 0.001 (0.015) |
| Economic tax | -0.024 (0.008)** | -0.011 (0.010) |  | -0.013 (0.009) | -0.008 (0.013) |
| Marijuana | 0.018 (0.003)*** | 0.016 (0.003)*** |  | 0.035 (0.005)*** | 0.030 (0.005)*** |
| Tobacco taxes | -0.040 (0.004)*** | -0.033 (0.005)*** |  | -0.026 (0.004)*** | -0.023 (0.005)*** |
| Constant | 2.609 (0.026)*** | 2.538 (0.037)*** |  | 2.812 (0.027)*** | 2.731 (0.033)*** |
|  |  |  |  |  |  |
| N | 1,050 | 1,050 |  | 1,050 | 1,050 |
| F-statistic | 3,404*** | 21385*** |  | 2,833*** | 612520*** |
| R2 (within) | 0.591 | 0.623 |  | 0.552 | 0.597 |

**p*<0.001; ***p*<0.01; **p*<0.05; †*p*<0.10

*Notes:* All models include fixed effects for states and, for each state-year observation, the sum of opioid policies, the percentage of the population that are immigrants, and the unemployment percentage.

^a^ Each domain is scaled from 0 to 1, where 0 is maximum conservative and 1 is maximum liberal observed.

^b^ Other domains from Grumbach (2018) include abortion, campaign finance, civil rights, education, housing and transportation, immigration, LGBT, public labor, and voting.

| **S1 Table 9. Percentage of adults ages 25–64 residing in states with policy domain scores more conservative than the national average during the 1994 to 2014 period** | | | |
| --- | --- | --- | --- |
| Policy Domain | Average score for each domain across all states and years 1994–2014 | Percent of adults ages 25–64 in 1994 and 2014 residing in states whose scores on each domain were below (i.e., more conservative than) the national average | |
|  |  | **1994** | **2014** |
| Criminal justice | 0.42 | 79.6 | 72.3 |
| Economic tax | 0.53 | 43.6 | 38.2 |
| Environment | 0.34 | 61.0 | 18.2 |
| Firearm safety | 0.28 | 28.9 | 54.6 |
| Health & welfare | 0.40 | 80.4 | 26.6 |
| Marijuana | 0.21 | 67.0 | 44.5 |
| Private labor | 0.51 | 37.4 | 51.4 |
| Tobacco tax | 0.81 | 88.2 | 21.8 |

Green = On average, the policy domain trended in a direction potentially beneficial for mortality

Red = On average, the policy domain trended in a direction potentially harmful for mortality

Purple = The policy domain was unrelated to mortality in the adjusted, all-cause mortality models (S1 Table 1)
